# Chaetocin Abrogates the Self-Renewal of Bladder Cancer Stem Cells via the Suppression of the KMT1A–GATA3–STAT3 Circuit

**DOI:** 10.3389/fcell.2020.00424

**Published:** 2020-06-17

**Authors:** Zhao Yang, Haifeng Wang, Nan Zhang, Tianying Xing, Wei Zhang, Guoqing Wang, Chong Li, Changyuan Yu

**Affiliations:** ^1^College of Life Science and Technology, Beijing University of Chemical Technology, Beijing, China; ^2^Department of Urology, Second Affiliated Hospital of Kunming Medical University, Kunming, China; ^3^Department of Urology, Xuanwu Hospital, Capital Medical University, Beijing, China; ^4^Department of Urology, Affiliated Hospital of Hebei University, Baoding, China; ^5^Department of Pathogenobiology, College of Basic Medical Science, Jilin University, Changchun, China; ^6^Core Facility for Protein Research, Institute of Biophysics, Chinese Academy of Sciences, Beijing, China

**Keywords:** bladder cancer, bladder cancer stem cells, KMT1A, chaetocin, target therapy

## Abstract

Bladder cancer stem cells (BCSCs) have the abilities of self-renewal, differentiation, and metastasis; confer drug resistance; and exhibit high tumorigenicity. We previously identified that the KMT1A–GATA3–STAT3 axis drives the self-renewal of BCSCs. However, the therapeutic effect of targeting KMT1A in BCSCs remains unknown. In this study, we confirmed that the expression of KMT1A was remarkably higher in BCSCs (3–5-fold) than those in bladder cancer non-stem cells or normal bladder epithelial cells. Among the six KMT1A inhibitors, chaetocin significantly suppressed the cell propagation (inhibition ratio: 65%–88%, IC_50_ = 24.4–32.5 nM), induced apoptosis (2–5-fold), and caused G1 phase cell cycle arrest (68.9 vs 55.5%) of bladder cancer (BC) cells, without influencing normal bladder epithelial cells. More importantly, chaetocin abrogated the self-renewal of BCSCs (inhibition ratio: 80.1%) via the suppression of the KMT1A–GATA3–STAT3 circuit and other stemness-related pathways. Finally, intravesical instillation of chaetocin remarkably inhibited the growth of xenograft tumors (inhibition ratio: 71–82%) and prolonged the survival of tumor-bearing mice (70 vs 53 days). In sum, chaetocin abrogated the stemness maintenance and tumor growth of BCSCs via the suppression of the KMT1A–GATA3–STAT3 circuit. Chaetocin is an effective inhibitor targeting KMT1A in BCSCs and could be a promising therapeutic strategy for BC.

## Introduction

Bladder cancer (BC) is a serious threat to public health and has one of the highest economically burdened tumor types worldwide (Leal et al., 2016; Wang H. et al., 2019). Based on the GLOBOCAN Cancer Statistics 2018, there were about 549,393 new cases of BC, which ranked first in urologic neoplasms. Additionally, more than 200,000 deaths were estimated in 2018 (Bray et al., 2018). Clinically, BC has three major types, which are transitional cell carcinoma (TCC), squamous cell carcinoma, and adenocarcinoma, in which TCC comprises >90% of BC patients (Wu et al., 1998).

Having distinct clinical characteristics and prognosis, BC is categorized into non-muscle invasive (NMI) and muscle invasive (MI; Kamat et al., 2016) types. Commonly, surgical electrocision, radical cystectomy, and bricker operation combined with chemotherapy are the primary treatments for BC patients (Lobo et al., 2017). In 2016, the United States Food and Drug Administration (FDA) approved the usage of immune checkpoint inhibitors atezolizumab (Cattrini and Boccardo, 2018), durvalumab (Brower, 2016), and avelumab (Vandeveer et al., 2016) in treating locally advanced or metastatic BC. However, the response rate of BC patients toward immune checkpoint inhibitors remained low (Rosenberg et al., 2016; Balar et al., 2017) and the recurrence rate of BC was high (Wu et al., 2016). Thus, it is of great importance to explore the mechanisms of BC tumorigenesis and novel therapeutic targets.

Serving as the cause of tumorigenesis, a factor of promoting tumor development and conferring drug resistance, cancer stem cells (CSCs) or cancer-initiating cells (CICs) were regarded as the promising target for tumor therapy (Zhou et al., 2009). Through high-throughput and large-scale compound screenings, Gupta et al. (2009) demonstrated that the inhibitory effect of salinomycin on breast CSCs was significantly higher than chemotherapy drug paclitaxel which could effectively reduce the growth and metastasis of breast cancer tumors. Besides strengthening anti-tumor immunity, catumaxomab exhibited significant anti-proliferation effects on ovarian CSCs via targeting EpCAM and achieved a positive outcome in clinical trials (Burges et al., 2007). Therefore, these findings highlighted the importance of targeting CSCs to combat cancer effectively.

Isolated via magnetic-activated cell sorting, bladder cancer stem cells (BCSCs), which expressed CD44v6^+^ and epithelial membrane antigen negative (EMA^–^), harbored high tumorigenicity and colony-forming and self-renewal capabilities (Yang and Chang, 2008). Besides, He et al. (2009) isolated BCSCs via the characterization of three biomarkers such as basal cell marker CK17 of normal urothelium, 67-kD laminin receptor (67LR), and absence of CEACAM6. These 67LR^+^CK17^+^CEACAM6^–^ BCSCs exhibited higher tumor formation capacity as compared to remaining BC cells (He et al., 2009). Furthermore, the range of biomarkers such as CD44 (Chan et al., 2009), CD90 (Volkmer et al., 2012), CK5 (Chan et al., 2009), CK14 (Volkmer et al., 2012), acetaldehyde dehydrogenase (ALDH; Su et al., 2010), ATP-binding cassette, sub-family G, member 2 (ABCG2; Ning et al., 2009), and aberrantly glycosylated integrin α3β1 (Li et al., 2016) were screened for the isolation of BCSCs.

Similar to normal bladder stem cells, BCSCs harbored the capabilities of high tumorigenicity, self-renewal, and differentiation to reestablish a heterogeneous hierarchy of BC (Volkmer et al., 2012). According to the recent research, the Hedgehog/GLI1 pathway (Chan et al., 2009), WNT/β-catenin pathway (Chan et al., 2009), and BMI1 (Chan et al., 2009) were involved in the self-renewal of BCSCs. Based on the biomarkers and self-renewal mechanisms of BCSCs, Chan et al. (2009) found that CD47 was widely expressed in BC cells and enriched specifically in BCSCs. Blocking CD47 enhanced the recognition and phagocytosis of BCSCs by macrophages (Chan et al., 2009). Our previous work had demonstrated that GALNT1 which was highly expressed in BCSCs promoted stemness maintenance of BCSCs. Cyclopamine, an Smoothened (SMO) inhibitor, significantly inhibited the abilities of self-renewal and tumor formation of BCSCs (Li et al., 2016). However, the reported biomarkers and therapeutic targets were not specific to BCSCs as they were expressed in normal bladder (stem) cells. Thus, it is necessary to identify the specific targets of BCSCs through massive screening of their inhibitors.

*KMT1A* encodes an evolutionarily conserved histone methyltransferase trimethylating histone H3 lysine 9 (H3K9me3), which led to transcriptional suppression (Bulut-Karslioglu et al., 2014). KMT1A participates in the regulation of embryonic development, cellular differentiation, cell cycle, and telomere length (Lee et al., 2011). Greiner et al. (2005) identified that the fungal metabolite chaetocin as the first inhibitor of lysine-specific histone methyltransferase, especially for the methyltransferase SU(VAR)3-9 both *in vitro* and *in vivo*. Firstly examined in tumor cells, chaetocin inhibited the proliferation of myeloma cells by inducing oxidative stress while sparing normal CD138^–^ patient bone marrow leukocytes, normal B cells, and neoplastic B-chronic lymphocytic leukemia (B-CLL) cells (Isham et al., 2007). Apart from that, Lakshmikuttyamma et al. (2010) reported that chaetocin decreased the cell proliferation and enhanced the apoptosis of acute myeloid leukemia (AML) cells by inhibiting KMT1A. Mechanically, chaetocin induced the expression of p15INK4B and E-cadherin via H3K9 demethylation (Lakshmikuttyamma et al., 2010). Moreover, Yokoyama et al. (2013) showed that knockdown of SUV39H1 or inhibition by chaetocin significantly inhibited the viability and migration of breast and colorectal cancer cells. Not only that, chaetocin demonstrated robust anti-cancer effects toward leukemia (Tran et al., 2013), non-small cell lung cancer (Liu et al., 2015), and hepatocellular carcinoma (Chiba et al., 2015).

In the previous study, we identified a novel self-renewal signaling, namely, KMT1A–GATA3–STAT3, which promoted the tumorigenicity and self-renewal maintenance of human BCSCs. Since BCSCs expressed significantly higher KMT1A as compared to bladder cancer non-stem cells (BCNSCs) or normal bladder cells, KMT1A could be regarded as a promising target of BCSCs. In this study, we demonstrated that chaetocin, as the inhibitor of KMT1A, could effectively suppress the self-renewal and tumorigenicity of BCSCs *in vitro* and *in vivo*.

## Materials and Methods

### Patient Tissues and Mice

Human primary normal bladder and BC tissues were obtained from The Second Affiliated Hospital of Kunming Medical University College (Kunming, China) with informed consent and approved by the Research Ethics Board at The Second Affiliated Hospital of Kunming Medical University. Flow cytometry cell sorting was conducted to sort BCSCs and BCNSCs. The freshly sorted BCSCs and BCNSCs were instantly applied for mRNA expression studies via quantitative real-time PCR (qRT-PCR) studies, protein expression analysis via Western blot (WB), and tumorsphere formation experiments. Besides, the sorted BCSCs were cultured in DMEM/F-12 medium supplemented with 20 ng/mL EGF, 20 ng/mL bFGF, 1% N2, and 2% B27, while BCNSCs were cultured in DMEM/F-12 medium supplemented with 15% FBS for 2–3 weeks until the number of cells was sufficient for WB, generation of xenograft, chromatin immunoprecipitation (ChIP), and DNase I digestion experiments. Detailed clinical information of the BC patients is concluded in Supplementary Table S1. The mice were maintained under standard conditions according to the institutional guidelines for animal care. All animal experiments were approved by the Animal Ethics Committee of The Second Affiliated Hospital of Kunming Medical University College (Kunming, China) and were carried out in compliance with the Animal Management Rules of the Ministry of Health of the People’s Republic of China.

### Cell Lines

Cell line EJ was obtained from the KeyGen BioTECH (KG046). Cell lines SV-HUC-1 and T24 were obtained from the American Type Culture Collection (CRL-9520 and HTB-4). BIU87 was obtained from the BaNa culture collection (BNCC341181). The cell lines were cultured in RPMI 1640 or DMEM (HyClone, SH30809.01 and SH30243.01) supplemented with 10% FBS (Gibco, A31608), 100 U/mL penicillin (SV30010), and 100 μg/mL streptomycin (SV30010). The cells were cultivated at 37°C with 5% CO_2_ in a humidified atmosphere. All cell lines were authenticated prior to use and tested for mycoplasma contamination routinely.

### Reagents and Antibodies

Chaetocin (S8068), AMI-1 (S7884), BIX-01294 (S8006), GSK343 (S7164), 3-deazaneplanocin (S7120), and UNC0631 (S8071) were purchased from Selleek. Horseradish peroxidase (HRP) labeled secondary antibody (LK2003) and β-actin (KM9001T) were purchased from Sungene Biotech. Antibodies such as KMT1A (ab12405), H3K9me3 (ab176916), and GATA3 (ab199428) were purchased from Abcam.

### Immunohistochemistry

Immunohistochemical staining was performed as previously described (Ye et al., 2014). Paraffin slices were dewaxed from xylene to water gradually, incubated with 3% H_2_O_2_ for 10 min at room temperature. Then, slices were washed by PBS for three times and sealed with 5% goat serum (Thermo Fisher Scientific, 16210064) diluted by PBS for 30 min, washed by PBS for three times, and dripped with KMT1A primary antibody and incubated at 4°C overnight. Finally, slices were washed by PBS for three times and incubated with appropriate HRP-conjugated secondary antibody for 1 h at 37°C. After three times of PBS washes, the substrate DAB was added for coloration. The staining scores of KMT1A were measured by multiplying the numerical score of the staining intensity (none = 1, weak = 2, moderate = 3, strong = 4) with the staining percentage (0–100%), resulting in an overall product score.

### Western Blot

Primary BC cells (tumor 1–3, #5–8, and peri-tumor 1–3, #5–8) and cell lines (SV-HUC-1, EJ, T24, and BIU87) were lysed using RIPA buffer [50 mmol/L Tris–HCl (pH 7.4), 150 mmol/L NaCl, 0.5% sodium deoxycholate, 0.1% SDS, 5 mmol/L EDTA, 2 mmol/L PMSF, and 1% Non-idet P-40] for 2 h. Proteins were separated using polyacrylamide gel electrophoresis and transferred to a nitrocellulose membrane (Millipore). The membranes were blocked using skim milk, probed by primary antibodies and HRP-conjugated secondary antibodies. The antibody bindings were detected using Pierce ECL Western Blotting Substrate (Thermo Fisher Scientific).

### PCR and qRT-PCR

RNA isolation kit (TIANGEN, S7522) and Reverse Transcriptase kit (TIANGEN, KR116-02) were used for total RNA extraction from cells and cDNA synthesis, respectively. The cDNA was used as the templates for qRT-PCR and quantitative analysis of the candidate genes, running in a Station One analyzer (Applied Biosystems). Primer sequences are listed in Supplementary Table S2. SYBR Green was used as the fluorescent probe. The housekeeping gene GAPDH was used as control to identify the relative expression level of the target genes. The fold change of differentially expressed genes in BCSCs compared with BCNSCs was calculated with the method of 2^–ΔΔCt^ (Yang et al., 2017).

### Cell Proliferation Assay

WST-1 kit (Beyotime, C0036L) colorimetric assay was used to measure the proliferation and viabilities of the cell lines (SV-HUC-1, BIU87, and T24) and primary BC cells (T1 and T2 and PT1 and PT2). The cells were seeded on a 96-well plate with a density of 3 × 10^3^ cells treated with 1% DMSO, 100 mM, AMI-1, 2.5 mM BIX-01294, 5–20 mM GSK343, 2.5 mM 3-deazaneplanocin, 50 nM chaetocin, and 1–5 mM UNC0631, respectively, for 24, 48, and 72-h incubations. At the specific time point, the supernatant was removed and 100 μL of 1640 medium containing 10 μL of WST-1 was added to each well for 2 h at 37°C. The absorbance at 450 nm was measured with a plate reader (Thermo Fisher Scientific, 1410101). The experiments were conducted three times independently.

In the IC_50_ measurement of chaetocin, cells were treated with different concentrations of chaetocin including 1.0, 2.5, 5.0, 7.5, 10.0, 25.0, 50.0, 75.0, and 100 nM for 48 h. The IC_50_ values were calculated using GraphPad Prism 5 software.

### Cellular Apoptosis Assay

The cells were cultured with/without 50 nM chaetocin for 48 h. The Annexin V-FITC kit (Beyotime, C1063) was used following the manufacturer’s instructions. The cells were washed twice with PBS, digested, collected, and resuspended in 195 μL binding buffer. Five microliter Annexin V-FITC and 10 μL propidium iodide (PI) were added, and the cells were incubated for 10 min at room temperature in the darkness. After the addition of 200 μL binding buffer, Annexin V-positive cells were analyzed using the FACSCalibur flow cytometry system (BD Biosciences, United States). The experiments were conducted three times independently.

### Cell Cycle Assay

BIU87 and T24 were cultured with/without 50 nM chaetocin for 48 h. The cells were fixed by pre-cooled 70% ethanol and incubated overnight at −20°C. Then, the cells were stained with 0.1 μg/mL RNase A and PI. The percentage of the cell population in each stage was measured by the flow cytometry FACSCalibur system and analyzed by CellQuest software (BD CellQuest Pro Software, BD Biosciences, United States).

### Flow Cytometry Cell Sorting

Human primary BC tissues were minced and digested using 20U type IV collagenase (Thermo Fisher Scientific, 17104019) and DNase I (GenStar, A216-101) at 37°C for 2 h. The cells were filtered to yield single cells and washed twice using PBS. The single-cell from the primary tissue and T24 and BIU87 cell lines were stained using FITC-conjugated BCMab1 (Li et al., 2014) and PE-conjugated anti-CD44 antibody (BD, 550989) for 30 min on ice. BCSCs (BCMab1^+^CD44^+^) and BCNSCs (BCMab1^–^CD44^–^) were analyzed and sorted by flow cytometry FACSAria II system.

### Tumorsphere Formation

BIU87 and T24 were seeded in an ultra-low-attachment surface 6-well plate (Corning) with a density of 5 × 10^3^ cells. The cells were maintained in DMEM/F12 medium supplemented with 20 ng/mL EGF, 20 ng/mL bFGF, 1% N2, and 2% B27.500 μL of fresh media was added every 3 days. The number of tumorspheres was counted 14 days after seeding. The whole assay was conducted four times independently.

### Chromatin Immunoprecipitation

Briefly, 10 million cancer cells were cross-linked with 1% formaldehyde and resuspended in lysis buffer. The cell lysate was sonicated on ice resulting in an average DNA fragment length of 500 bp. After centrifugation, immunoprecipitation was performed in ChIP dilution buffer overnight in the presence of IgG, H3K9me3, and GATA3 antibodies with agitation. The protein A agarose/Salmon Sperm DNA (Merck Millipore) slurry was added and incubated for 2 to 4 h at 4°C with agitation. The antibody-agarose complex was centrifuged and washed five times, and the immunoprecipitated fraction was eluted. The cross-linking was reversed by incubation at 65°C for 4 h. The DNA was recovered by phenol/chloroform extraction and precipitated, and the abundance of specific sequence was measured by qRT-PCR using the corresponding primer sequences (Supplementary Table S2).

### DNase I Digestion Assay

Cell nuclei were isolated and lysed for DNase I digestion assay as described (Yang et al., 2017). After digestion at 37°C for 5 min, total DNA was extracted to perform PCR assays using promoter-specific primers (Supplementary Table S2).

### Generation of Xenografts

NOD/SCID mice were obtained from the Animal Center of the Chinese Academy of Medical Sciences (Beijing, China). In order to generate xenograft, BIU87, T24, and primary BC cells (samples #14 and #15) with a density of 5 × 10^6^ cells were injected subcutaneously into the NOD/SCID mice (*n* = 10). After 1 week, the volume of tumors was observed and the mice were grouped and administered intraperitoneally with DMSO or chaetocin at a dose of 0.3 mg/kg every 3 days for 8 weeks. The volume of tumors was measured per 3 days, *V* = (π/6) (*a* × *b* × *c*). Mice were sacrificed after 8 weeks.

### Statistical Analysis

Student’s *t* test was used to compare the mean values of two groups. In the gene expression and survival analysis, the average of gene expression was first calculated. BC samples expressing higher levels of *KMT1A* than the average were defined as *KMT1A* high group and the remaining samples as *KMT1A* low group. The overall survival of each group was calculated by a Kaplan–Meier analysis, and the difference between those two groups was examined using the log-rank test. The difference with *P* < 0.05 was regarded as significant difference.

## Results

### KMT1A Is Highly Expressed in Bladder Cancer

**FIGURE 1:**
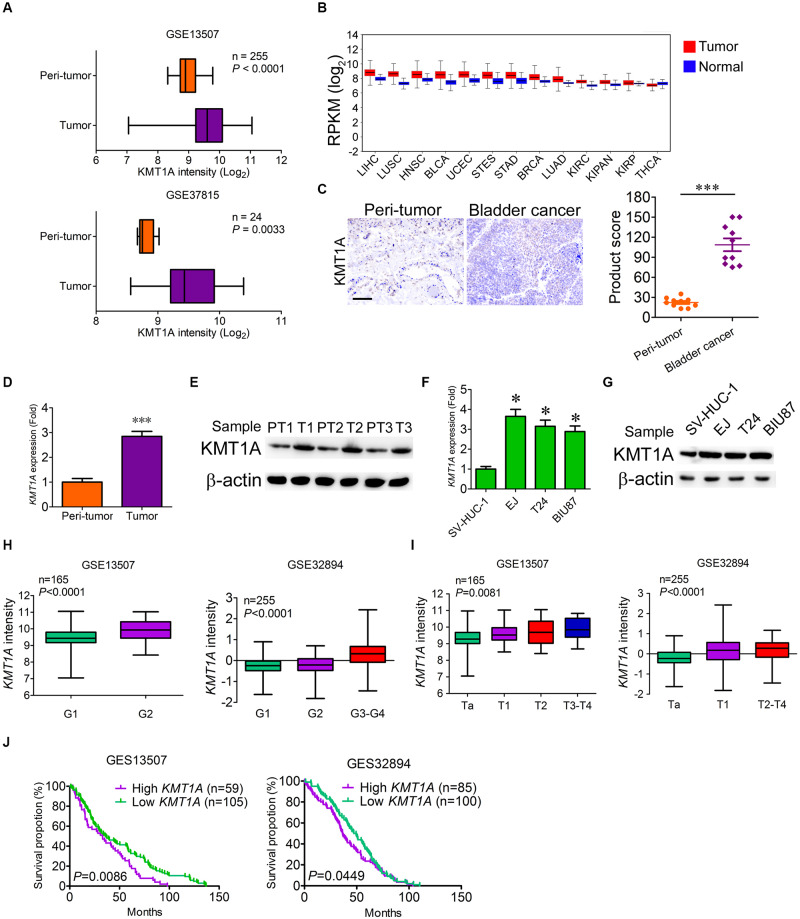
KMT1A is highly expressed in bladder cancer. **(A)** The expression of *KMT1A* was analyzed according to the data from Bae’s cohort (GSE13507) and Kim’s cohort (GSE37815). **(B)** The expression of *KMT1A* was analyzed according to the TCGA database. *KMT1A* was highly expressed in ESCA, STES, STAD, LUSC, HNSC, BLCA, and LIHC. BLCA, bladder urothelial carcinoma; BRCA, breast invasive carcinoma; COAD, colon adenocarcinoma; COADREAD, colorectal adenocarcinoma; ESCA, esophageal carcinoma; HNSC, head and neck squamous cell carcinoma; KIPAN, pan-kidney cohort (KICH + KIRC + KIRP); KIRC, kidney renal clear cell carcinoma; KIRP, kidney renal papillary cell carcinoma; LAML, acute myeloid leukemia; LIHC, liver hepatocellular carcinoma; LUAD, lung adenocarcinoma; LUSC, lung squamous cell carcinoma; OV, ovarian serous cystadenocarcinoma; READ, rectum adenocarcinoma; STAD; stomach adenocarcinoma, STES, stomach and esophageal carcinoma; THCA, thyroid carcinoma; UCEC, uterine corpus endometrial carcinoma. **(C)** The expression of KMT1A was higher in BC samples than those in peri-tumors as assessed by IHC (*n* = 10). KMT1A staining was measured by multiplying the numerical score of the staining intensity (none = 1, weak = 2, moderate = 3, strong = 4) with the staining percentage (0%–100%), resulting in an overall product score (*n* = 4), Student’s t test. Scale bar = 50 μm. **(D)** *KMT1A* was highly expressed in primary BC tissues compared to those in normal bladder tissues by qRT-PCR (*n* = 5), Student’s *t* test. **(E)** The expression of KMT1A was determined in primary normal bladder and BC tissues by WB. β-Actin served as a loading control. **(F)** *KMT1A* was highly expressed in EJ, T24, and BIU87 compared to that in SV-HUC-1 by qRT-PCR (*n* = 3), Student’s *t* test. **(G)** The expression of KMT1A was determined in bladder epithelial cell line SV-HUC-1 and BC cell lines EJ, T24, and BIU87 by WB. β-Actin served as a loading control. **(H,I)**. The expression of *KMT1A* is positively correlated with the grade and stage of BC patients based on GSE13507 and GSE32894 cohorts. **(J)** Kaplan–Meier curves compared the overall survival of BC patients who expressed high or low levels of *KMT1A* based on GSE13507 and GSE32894 cohorts. *n*, patient number. Data are presented as mean ± SD. ^∗^*P* < 0.05, ^∗∗∗^*P* < 0.001.

Our previous study showed that histone methyltransferase KMT1A promoted self-renewal of BCSCs via the KMT1A–GATA3–STAT3 signaling pathway (Yang et al., 2017). To verify whether KMT1A is a candidate for targeted therapy of BC, the expression of KMT1A was first examined in tumor and normal/peri-tumor tissues from BC patients. Based on the analysis of microarray data from GEO datasets (GSE13507 and GSE37815), *KMT1A* was highly expressed in tumor tissues as compared to peri-tumor tissues (Figure 1A). Based on the data extracted from The Cancer Genome Atlas (TCGA) database^1^, *KMT1A* was also highly expressed in esophageal carcinoma, stomach and esophageal carcinoma, stomach adenocarcinoma, lung squamous cell carcinoma, head and neck squamous cell carcinoma, bladder urothelial carcinoma, and liver hepatocellular carcinoma (Figure 1B). In Immunohistochemistry (IHC) analysis, the staining scores of KMT1A were significantly elevated in tumor tissues as compared to peri-tumor tissues from BC patients (Figure 1C).

To ascertain the difference of KMT1A expression in BC cells and peri-tumor cells, qRT-PCR and WB were carried out. The results showed that the expression of KMT1A in BC cells was 2.85-fold higher than peri-tumor cells in both the mRNA and protein levels (Figures 1D,E). Similarly, the expression of KMT1A was significantly enhanced in BC cell lines EJ (3.65-fold), T24 (3.15-fold), and BIU87 (2.89-fold) as compared to the immortalized human bladder epithelial cells SV-HUC-1 in both mRNA and protein levels (Figures 1F,G). Furthermore, the expression of *KMT1A* was positively correlated with the grade and stage in Bae’s cohort (GSE13507) and Hoglund’s cohort (GSE32894; Figures 1H,I). In the survival analysis, the average overall survival time of *KMT1A* high group and *KMT1A* low group in GSE13507 was 36.3 and 49.1 months, respectively. Moreover, the average overall survival time of *KMT1A* high group and *KMT1A* low group in GSE32894 was 41.0 and 46.5 months, respectively. The results indicated that BC patients expressing higher levels of *KMT1A* had a shorter mean survival time than did the patients expressing lower levels of *KMT1A* in both GSE13507 (*P* = 0.0086) and GSE32894 (*P* = 0.0449) cohorts (Figure 1J). Taken together, the expression of KMT1A in BC cells is significantly higher than normal bladder epithelial cells and peri-tumor cells. Also, KMT1A is positively associated with tumor grade and stage, but negatively correlated with prognosis.

### Chaetocin Selectively Inhibits the Proliferation of Bladder Cancer Cells

**FIGURE 2:**
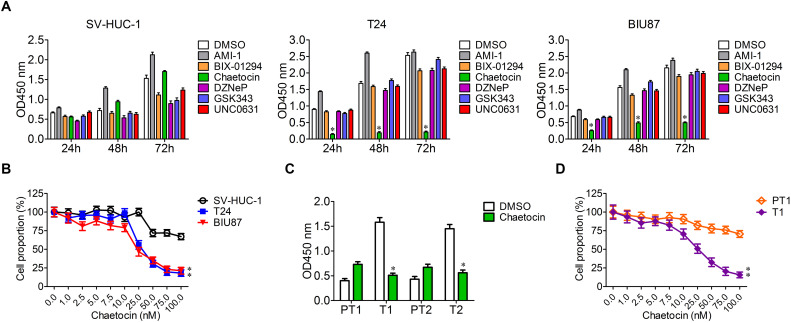
Chaetocin inhibits the proliferation of bladder cancer cells *in vitro*. **(A)** Chaetocin inhibited BC cell growth in a time-dependent manner. Six histone methyltransferase inhibitors, including AMI-1, BIX-01294, chaetocin, DZNeP, GSK343, and UNC0631, were selected for cell proliferation experiment. **(B)** Chaetocin inhibited primary BC cell growth in a dose-dependent manner. **(C)** Chaetocin suppressed the proliferation of primary BC cells (T1 and T2). **(D)** Chaetocin inhibited the propagation of primary BC cells (T1) in a dose-dependent manner. The whole assay was repeated three times. Data are presented as mean ± SD, Student’s *t* test. ^∗^*P* < 0.05, ^∗∗^*P* < 0.01.

To determine the specific and effective inhibitors to BC, six histone methyltransferase inhibitors including AMI-1, BIX-01294, chaetocin, DZNeP, GSK343, and UNC0631 were selected for cell proliferation experiment. SV-HUC-1, T24, and BIU87 cells were cultured in the presence of the six inhibitors, and cell proliferation was analyzed with the method of cell counting kit-8 at 24, 48, and 72 h. The growth of SV-HUC-1, T24, and BIU87 cells was not affected by AMI-1, BIX-01294, DZNeP, GSK343, and UNC0631 (Figure 2A). Interestingly, chaetocin significantly inhibited the proliferation of both T24 (inhibition rate: 88%) and BIU87 (inhibition rate: 69%) cells while sparing SV-HUC-1 cells (Figure 2A). Since chaetocin possessed significant anti-proliferation effects as compared to other inhibitors, different concentrations of chaetocin were administered to SV-HUC-1, T24, and BIU87 to determine IC_50_ for future experiments. As compared to SV-HUC-1 cells (IC_50_: 156.5 nM), chaetocin significantly inhibited the growth of T24 and BIU87 cells with an IC_50_ of 26.8 and 32.5 nM, respectively (Figure 2B). When SV-HUC-1, BIU87, and T24 were treated with 50 nM chaetocin, the cell proportion of both T24 and BIU87 were less than 50%, while the cell proportion of SV-HUC-1 remained high (Figure 2B). Similarly, chaetocin significantly inhibited the growth of primary tumor cells (T1 and T2, repression rate: 65%), but not affecting the proliferation of peri-tumor cells (PT1 and PT2), which were isolated from BC patients (Figure 2C). Besides, chaetocin decreased the cell proliferation of primary tumor cells (T1) in a dose-dependent manner with a lower IC_50_ (24.4 nM) while sparing peri-tumor cells (PT1, IC_50_: 613.2 nM; Figure 2D). In summary, chaetocin significantly suppresses the proliferation of BC cells (inhibition ratio: 65–88%, IC_50_ = 24.4–32.5 nM) without causing an inhibitory effect on bladder epithelial or peri-tumor cells.

### Chaetocin Induces Apoptosis and G1 Phase Cell Cycle Arrest of Bladder Cancer Cells

**FIGURE 3:**
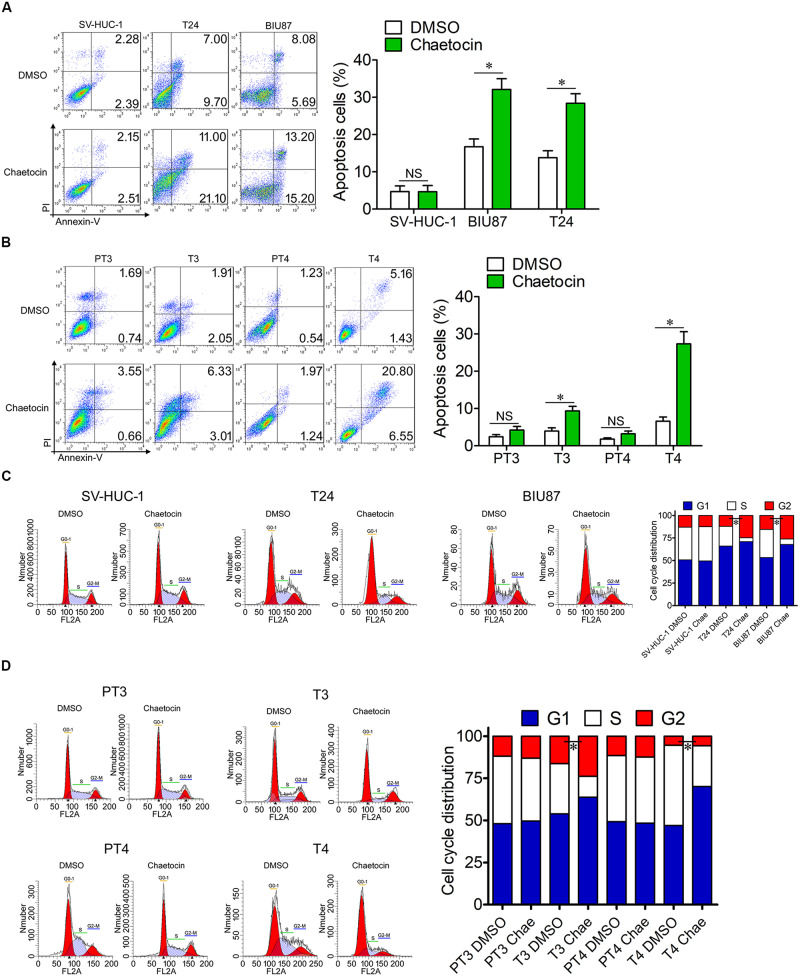
Chaetocin induces apoptosis and G1 phase cell cycle arrest of bladder cancer cells *in vitro*. Chaetocin induced apoptosis of **(A)** T24 and BIU87 cell lines and **(B)** primary BC cells (T3 and T4). The apoptosis cells were stained with Annexin V-FITC and PI staining and detected by FACSCalibur. Chaetocin induced G1 phase cell cycle arrest in panel **(C)** both BC cell lines and **(D)** primary BC cells (T3 and T4). The cell cycle distributions of cells were determined after 70% ethanol fixation and PI staining. The whole assay was repeated three times. Data are presented as mean ± SD, Student’s *t* test. ^∗^*P* < 0.05.

Tumor cells are characterized by the capabilities of anti-apoptosis and uncontrolled cell division. To investigate the effect of chaetocin on apoptosis and cell cycle of SV-HUC-1, T24, and BIU87 cells, the cells were treated with 32.5 nM chaetocin for 48 h. In cellular apoptosis assays, chaetocin did not cause the apoptosis of SV-HUC-1 cells. However, chaetocin significantly induced apoptosis of T24 (28.4%) and BIU87 cells (32.1%), which are significantly higher than those of DMSO group (apoptosis rate: 4.7%, Figure 3A). To examine the apoptotic activity of chaetocin toward primary BC cells (T3 and T4) and peri-tumor cells (PT3 and PT4) which are derived from BC patients, the cells were treated with 24.4 nM chaetocin and incubated for 48 h. The results showed that chaetocin exhibited 2–5-fold higher apoptotic activities than the DMSO group did toward primary tumor cells (Figure 3B). However, chaetocin exhibited no apoptotic effects toward peri-tumor cells (Figure 3B).

To further identify the effect of chaetocin on the cell cycle, the proportion of cells in different cell cycle distributions was measured through PI staining. In cell cycle assays, chaetocin did not affect the cell cycle distribution of SV-HUC-1 cells. However, both T24 and BIU87 cells underwent G1 phase cell cycle arrest, indicating that the number of mitotic cells remarkably decreased under the treatment of chaetocin (Figure 3C). Similarly, chaetocin significantly reduced the number of primary tumor cells (T3 and T4) in the S phase and increased in the G1 phase, suggesting that chaetocin induced G1 phase cell cycle arrest in primary tumor cells (68.9 vs 55.5%). However, chaetocin did not change the cell cycle distribution of peri-tumor cells (48.7 vs 49.1%; PT3 and PT4; Figure 3D). Taken together, chaetocin significantly induces apoptosis and G1 phase cell cycle arrest of BC cells without affecting bladder epithelial or peri-tumor cells.

### Chaetocin Suppresses the Expression of Stemness-Related Pathways in BCSCs

**FIGURE 4:**
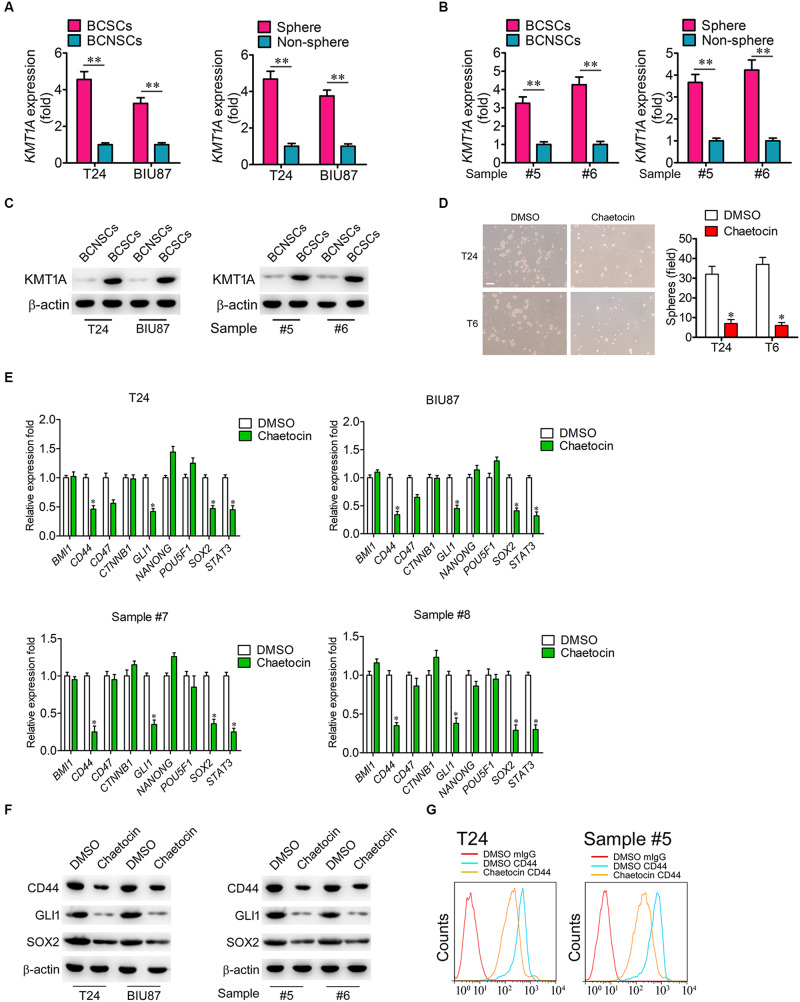
Chaetocin suppress the expression of stemness-related genes in BCSCs. The expression of *KMT1A* was examined in BCSCs, BCNSCs, and tumorspheres, non-tumorspheres of **(A)** T24 and BIU87 cells and **(B)** primary BC samples (#5 and #6) through qRT-PCR analysis, *n* = 4, Student’s *t*-test. Non-sphere: BC cells that failed to form tumorspheres. **(C)** The protein expression of KMT1A in BCSCs, BCNSCs, and tumorspheres, non-tumorsphere of T24, BIU87 cells, and primary BC samples (#5 and #6) through WB. β-Actin served as a loading control. **(D)** Chaetocin significantly inhibited the tumorsphere formation of BCSCs derived from T24 and primary BC samples (T6). The number of tumorspheres was counted in five independent fields/well after 2 weeks of cultivation. Scale bar = 100 μm. **(E)** The expression levels of *BMI1*, *CD44*, *CD47*, *CTNNB*1, *GLI1*, *NANOG*, *POU5F1*, *SOX2*, and *STAT3* in BCSCs derived from T24, BIU87, and primary BC samples (#7 and #8) under the treatment of DMSO or chaetocin through qRT-PCR analysis. **(F)** The protein expression of CD44, GLI1, and SOX2 in BCSCs isolated from T24 and BIU87 cells and primary BC samples (#5 and #6) through WB. β-Actin served as a loading control. **(G)** Chaetocin decreased the parentage of CD44^+^ cells in BCSCs isolated from T24 and primary BC sample #5. The whole assay was repeated three times. Data are presented as mean ± SD, Student’s *t* test. ^∗^*P* < 0.05, ^∗∗^*P* < 0.01.

According to our previous study, KMT1A is a candidate biomarker of BCSCs (Yang et al., 2017). To investigate whether chaetocin could inhibit the self-renewal and stemness of BCSCs, the expression of KMT1A was first examined in BCSCs and BCNSCs. In qRT-PCR expression studies, the expression of *KMT1A* in BCSCs (BCMab1^+^CD44^+^) and tumorsphere cells was significantly higher than those of BCNSCs (BCMab1^–^CD44^–^) and non-tumorsphere cells of T24, BIU87 cell, and primary BC samples (3–5 fold) (#5 and #6) (Figures 4A,B). Similarly, the protein expression level of KMT1A was significantly expressed in BCSCs rather than BCNSCs of T24 and BIU87 cell lines and primary BC samples (#5 and #6; Figure 4C). Based on these results, the effect of chaetocin on the tumorsphere formation ability of BCSCs was conducted. In the serum-free condition, BCSCs of T24 and BIU87 cell lines and primary BC samples (#5 and #6) formed tumorspheres distinctly. However, the number and volume of tumorspheres decreased significantly under the treatment of chaetocin (inhibition ratio: 80.1%, Figure 4D).

To further investigate whether chaetocin could inhibit the expression of stemness-related genes, BCSCs of T24, BIU87, and primary BC samples (#7 and #8) were treated with chaetocin in a serum-free culture medium for 48 h and subjected to qRT-PCR, WB, and FACS studies. As compared to DMSO treatment, chaetocin significantly suppressed the expression of CD44, GLI1, SOX2, and STAT3 in BCSCs and primary BC samples, in both mRNA (Figure 4E) and protein levels (Figures 4F,G). In summary, KMT1A is highly expressed in BCSCs and tumorspheres. Also, chaetocin inhibits the self-renewal of BCSCs via the suppression of stemness-related genes in BCSCs.

### Chaetocin Targets KMT1A–GATA3–STAT3 Signaling in BCSCs

**FIGURE 5:**
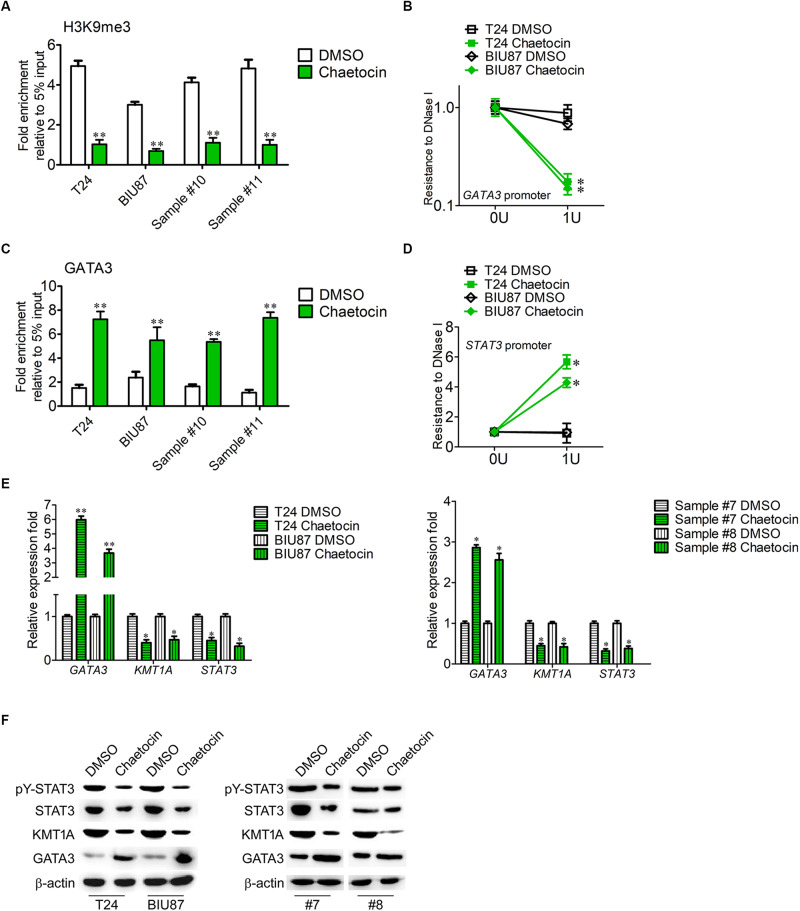
Chaetocin targets KMT1A–GATA3–STAT3 signaling in BCSCs. **(A)** ChIP analysis of the *GATA3* promoter using IgG and H3K9me3 antibodies in BCSCs derived from T24, BIU87, and primary BC samples (#7 and #8) under the treatment of DMSO or chaetocin. The enrichment of the *GATA3* promoter was detected by qRT-PCR. **(B)** Chaetocin decreased the resistance to DNase I digestion at the *GATA3* locus. **(C)** ChIP analysis of the *STAT3* promoter using IgG and GATA3 antibodies in BCSCs derived from T24, BIU87, and primary BC samples (#7 and #8) under the treatment of DMSO or chaetocin. The enrichment of the *STAT3* promoter was detected by qRT-PCR. **(D)** Chaetocin increased the resistance to DNase I digestion at the *STAT3* locus. **(E)** The expression levels of *GATA3*, *KMT1A*, and *STAT3* in BCSCs derived from T24, BIU87, and primary BC samples (#7 and #8) under the treatment of DMSO or chaetocin, through qRT-PCR analysis. **(F)** The protein expression of pY-STAT3, STAT3, KMT1A, and GATA3 in BCSCs isolated from T24, BIU87, and primary BC samples (#7 and #8) under the treatment of DMSO or chaetocin, through WB. β-Actin served as a loading control. The whole assay was repeated four times. Data are presented as mean ± SD, Student’s *t* test. ^∗^*P* < 0.05, ^∗∗^*P* < 0.01.

KMT1A–GATA3–STAT3 signaling is a novel self-renewal pathway of human BCSCs (Yang et al., 2017). To determine whether chaetocin targets the KMT1A–GATA3–STAT3 pathway in BCSCs, ChIP and DNase I resistance experiments were conducted. In ChIP analysis, the antibody that specifically binds to H3K9me3 was used to capture *GATA3* gene in BCSCs isolated from T24, BIU87, and primary BC samples (#10 and #11). Chaetocin significantly decreased the occupation of H3K9me3 modification on the promoter of *GATA3* in BCSCs as compared to DMSO treatment (Figure 5A). In DNase I digestion assay, chaetocin remarkably enhanced the chromatin accessibility of the *GATA3* locus in BCSCs (Figure 5B). Furthermore, the antibody that specifically binds to GATA3 was used to capture *STAT3* gene in BCSCs isolated from T24, BIU87, and primary BC samples (#10 and #11). In ChIP analysis, chaetocin significantly increased the occupation of GATA3 modification on the promoter of *STAT3* in BCSCs as compared to DMSO treatment (Figure 5C). In DNase I digestion assay, chaetocin remarkably enhanced the chromatin inaccessibility of the *STAT3* locus in BCSCs (Figure 5D). These experimental results showed that chaetocin reduced H3K9me3 modifications on *GATA3* loci and enhanced the chromatin accessibility of the *GATA3* but not *STAT3* promoter.

To demonstrate that chaetocin affects the activation of the KMT1A–GATA3–STAT3 pathway in BCSCs, the expressions of KMT1A, GATA3, and STAT3 were measured by qRT-PCR and WB under the treatment of DMSO and chaetocin. As compared to DMSO treatment, chaetocin significantly upregulated the expression of *GATA3* and downregulated the expression of KMT1A and STAT3, in both mRNA (Figure 5E) and protein (Figure 5F) levels. Taken together, chaetocin inhibits self-renewal of BCSCs by suppressing the KMT1A–GATA3–STAT3 signaling pathway.

### Chaetocin Suppresses the Tumor Growth of Bladder Cancer *in vivo*

**FIGURE 6:**
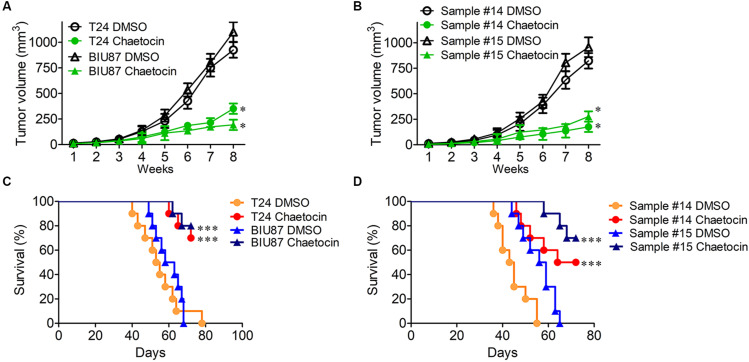
Chaetocin suppresses the tumor growth of bladder cancer *in vivo*. Chaetocin significantly suppressed the tumor growth of **(A)** T24 and BIU87 cells and **(B)** primary BC cells (sample #14 and #15) in xenograft mice (*n* = 10). Mice bearing tumor cells of T24, BIU87, and primary BC cells (sample #14 and #15) were administered intraperitoneally with DMSO or chaetocin two times per week. The volume of xenografts was measured every 3 days. Chaetocin significantly enhanced the survival of xenograft mice transplanted with **(C)** T24, BIU87 cells, and **(D)** primary BC cells (samples #14 and #15, *n* = 10). Data are expressed as mean ± SD. ^∗^*P* < 0.05, ^∗∗∗^*P* < 0.001.

The mouse xenograft BC models were established by subcutaneous injection of T24 and BIU87 cells and primary BC cells (#14 and #15) into the back of nude mice, respectively. One week later, BC xenografts could be observed on mice. When the diameter of tumor reached 3–5 mm, the mice were categorized into DMSO group and chaetocin group. Every 3 days, 0.3 mg/kg chaetocin was intraperitoneally injected into mice and the size of the tumor was measured. Eight weeks later, mice were sacrificed and the therapeutic effect was evaluated. As compared to DMSO treatment, chaetocin significantly inhibited the tumor growth of T24, BIU87, and primary BC cells (#14 and #15) *in vivo* (inhibition ratio: 71–82%) (Figures 6A,B). Furthermore, chaetocin significantly prolonged the lifespan of mice bearing BC tumors as compared to DMSO in the survival rate analysis (70vs 53 days) (Figures 6C,D). Taken together, chaetocin inhibits the tumor growth in the xenograft model and effectively prolongs the lifespan of mice suffering from BC.

## Discussion

There have been no major breakthroughs or advances in the treatment of BC in the past 30 years. Although traditional chemoradiotherapy can prolong the prognosis of patients with BC, it does not fundamentally solve the clinical queries of high recurrence (Kamat et al., 2016). Bladder cancer stem cells play a critical role in tumor initiation, metastasis, progression, and conferring of drug resistance. It expresses range of biomarkers signifying the stemness which are CD44 (Chan et al., 2009), CD90 (Volkmer et al., 2012), 67LR (He et al., 2009), CK5 (Chan et al., 2009), CK14 (Volkmer et al., 2012), CK17 (He et al., 2009), ALDH (Su et al., 2010), ABCG2 (Ning et al., 2009), aberrantly glycosylated integrin α3β1 (Li et al., 2014) and OV6 (Wang K.J. et al., 2019). Not only that, BCSCs activate the GLI1/Hedgehog pathway (Chan et al., 2009; Li et al., 2016), WNT/β-catenin axis (Gao et al., 2017), and KMT1A–GATA3–STAT3 circuit (Yang et al., 2017) to maintain the self-renewal properties. The emergence of BCSCs shed light on the target therapy of BC. Since KMT1A is significantly expressed in BCSCs rather than BCNSCs, KMT1A serves as a promising target for BC therapy. Herein, we identified chaetocin, an inhibitor of KMT1A, which inhibited the proliferation, induced apoptosis, and caused the G1 phase cell cycle arrest of BCSCs *in vitro*. Furthermore, chaetocin abrogated the self-renewal of BCSCs via the suppression of the stemness-related pathway and tumor growth *in vivo*.

Although chemotherapy such as cisplatin and gemcitabine shrunk the tumors, tumor cells tend to develop chemoresistance and implicated tumor relapse (Zhang et al., 2012; Seo et al., 2014). Kurtova et al. (2015) identified that the recruitment of the quiescent label-retaining pool of BCSCs and the release of prostaglandin E2 contributed to chemoresistance and the regeneration of BCSCs. To circumvent these problems, Celebrex effectively reversed BCSC activities and tumor recurrence besides prolonging the overall survival of tumor-bearing mice by blocking the PGE2 and COX2 signaling pathways (Kurtova et al., 2015). In addition, Chan et al. (2009) identified that CD47 was highly expressed in BCSCs and negatively regulated anti-tumor immunity. Anti-CD47 treatment not only increased the phagocytosis of macrophage but also inhibited the metastasis of BC *in vivo* (Chan et al., 2009). Besides, Zhu et al. (2016) showed that iG2, a novel Hedgehog (Hh) inhibitor from *Streptomyces roseofulvus*, suppressed the self-renewal and tumorigenesis of BCSCs by inhibiting the Hh signaling pathway (Zhu et al., 2016). These studies demonstrated the importance of targeting BCSCs and their regulation of signaling pathways in achieving effective treatment of BC.

To identify a more specific target of BCSCs for precision medicine of BC, our previous study demonstrated that KMT1A–GATA3–STAT3 triggers the self-renewal of BCSCs and KMT1A is a specific target of BCSCs (Yang et al., 2017). As a histone methyltransferase trimethylating H3K9me3, KMT1A participates in the regulation of embryonic development, cellular differentiation, cell cycle, and telomere length (Lee et al., 2011). In recent years, KMT1A has contributed significantly in the tumorigenesis of myeloma, leukemia (Tran et al., 2013), breast cancer (Yokoyama et al., 2013), colorectal cancer (Yokoyama et al., 2013), non-small cell lung cancer (Liu et al., 2015), and hepatocellular carcinoma (Chiba et al., 2015); thus, it serves as a potential therapeutic target in cancer treatment. Among six histone methyltransferase inhibitors, we identified that chaetocin exerted robust anti-cancer *in vitro* and *in vivo* by inhibiting tumor cell proliferation and inducing apoptosis and G1 phase cell cycle arrest via the suppression of the KMT1A–GATA3–STAT3 circuit and other stemness-related pathways. In concordance with our studies, various researches elucidated the potential of chaetocin as a cancer therapeutic agent. Chaetocin induced apoptosis through the regulation of the mitochondria intrinsic pathway and the inhibition of autophagy on cancerous cells (Jung et al., 2016; Han et al., 2017). Furthermore, chaetocin generates reactive oxygen species which damaged gastric cancer cells via the suppression of the PI3K/AKT tumor progression pathway (Wen et al., 2019). Although exhibiting promising results, some BC cells acquired resistance toward chaetocin and not all tumor-bearing mice survived under the treatment of chaetocin. These data suggested that BCSCs are highly heterogeneous (Ohishi et al., 2015). Overall, chaetocin is a potential therapeutic agent in treating BCSCs via the suppression of KMT1A.

## Conclusion

In conclusion, our findings demonstrate that chaetocin abrogated the stemness maintenance and tumor formation of BCSCs via the suppression of the KMT1A–GATA3–STAT3 circuit, which lays a theoretical foundation for an effective combination therapy strategy of BC. The heterogeneity of BCSCs and the potential resistance mechanisms toward chaetocin treatment should be further investigated for a more successful treatment of BC.

## Data Availability Statement

Publicly available datasets were analyzed in this study. This data can be found as follows: Three datasets were obtained from the Gene Expression Omnibus (GEO) database (GSE13507, https://www.ncbi.nlm.nih.gov/geo/query/acc.cgi?acc=GSE13507, GSE32894, https://www.ncbi.nlm.nih.gov/geo/query/acc.cgi?acc=GSE32894, and GSE37815, https://www.ncbi.nlm.nih.gov/geo/query/acc.cgi?acc=GSE37815). Additionally, the expression of *KMT1A* in tumor and normal tissues was obtained from the Cancer Genome Atlas (TCGA) database (http://firebrowse.org/).

## Ethics Statement

The studies involving human participants were reviewed and approved by the Research Ethics Board at The Second Affiliated Hospital of Kunming Medical University. The patients/participants provided their written informed consent to participate in this study. The animal study was reviewed and approved by the Animal Ethics Committee of The Second Affiliated Hospital of Kunming Medical University (Kunming, China) and were carried out in compliance with the Animal Management Rules of the Ministry of Health of the People’s Republic of China.

## Author Contributions

ZY designed the experiments. ZY and NZ performed the experiments, analyzed the data, and wrote the manuscript. HW, TX, and WZ prepared the samples and performed the experiments. GW and CY revised the manuscript. ZY and CL initiated the study and organized, designed, and revised the manuscript.

## Conflict of Interest

The authors declare that the research was conducted in the absence of any commercial or financial relationships that could be construed as a potential conflict of interest.

## Footnotes

1 http://firebrowse.org/
