# Supplementary material for: Chaetocin Abrogates the Self-Renewal of Bladder Cancer Stem Cells via the Suppression of the KMT1A–GATA3–STAT3 Circuit
Source: Front Cell Dev Biol. 2020 Jun 17;8:424. doi: 10.3389/fcell.2020.00424 (PMC7311639; doi:10.3389/fcell.2020.00424)
Supplement: Supplementary file 1 [file Table_1.pdf]

Supplementary Table S1. Clinical characteristics of the bladder carcinoma patients.

Patient ID#	Patient age (years)	Sex	Stage (TNM classification*)	Grade	Surgery	Primary/Recurrent
1	56	M	T1N0M0	high	TURBT	Primary
2	72	F	T2N0M0	high	RC	Primary
3	65	M	T3N0M0	high	RC	Primary
4	59	M	T1N0M0	high	TURBT	Primary
5	43	M	T1N0M0	high	TURBT	Primary
6	45	M	T2aN0M0	low	RC	Primary
7	46	F	T2N0M0	high	RC	Primary
8	57	M	T1N0M0	high	TURBT	Primary
10	62	M	T2aN0M0	low	RC	Primary
11	66	M	T1N0M0	high	TURBT	Primary
12	41	F	T2aN0M0	low	RC	Primary
13	69	M	T3N0M0	high	RC	Primary
14	58	M	T1N0M0	high	TURBT	Primary
15	47	M	T2N0M0	high	RC	Primary

#The samples were collected randomly and objectively. Only the samples possessed the enough number of BCSCs for the quantitative real-time PCR (qRT-PCR), western blot (WB), chromatin immunoprecipitation (ChIP), etc. experiments were recorded.

∗The TNM cancer staging system was designed to gauge the extent of cancer in a patient's body. T describes the size of the tumor and whether it has invaded nearby tissue, N describes regional lymph nodes that are involved, and M describes distant metastasis (spread of cancer from one body part to another). NA, not available. TURBT: transurethral resection of bladder tumor, RC: radical cystectomy.

Supplementary Table S2. PCR primer sequences for selected genes.

Gene	Forward primer	Reverse primer	Application
GAPDH	AAGGTGAAGGTCGGAGTCAA	GGAAGATGGTGATGGGATTT	RT-PCR
KMT1A	ATGCCGCCTACTATGGCAAC	AAAGTTGGAGTCCATGCGGG	RT-PCR
BMI1	CCACCTGATGTGTGTGCTTTG	TTCAGTAGTGGTCTGGTCTTGT	RT-PCR
CD44	CTGCCGCTTTGCAGGTGTA	CATTGTGGGCAAGGTGCTATT	RT-PCR
CD47	AGAAGGTGAAACGATCATCGAGC	CTCATCCATACCACCGGATCT	RT-PCR
CTNNB1	ACAACTGTTTTGAAAATCCA	CGAGTCATTGCATACTGTCC	RT-PCR
GLI1	TCCTTTATTATCAGGAAACAG	GAGTAGGGAATCTCATCCAT	RT-PCR
NANOG	TACCTCAGCCTCCAGCAGA	CCTCCAAGTCACTGGCAG	RT-PCR
POU5F1	GACAACAATGAAAATCTTCAGGAGA	TTCTGGCGCCGGTTACAGAACCA	RT-PCR
SOX2	ATGCACCGCTACGACGTGA	CTTTTGCACCCCTCCCATTT	RT-PCR
STAT3	ACCAGCAGTATAGCCGCTTC	GCCACAATCCGGGCAATCT	RT-PCR
GATA3	ACTTCCCCAAGAACAGCTCG	GTGGTGTGGTCCAAAGGACA	RT-PCR
GAPDH	TACTAGCGGTTTTACGGGCG	TCGAACAGGAGGAGCAGAGAGCGA	CHIP
GATA3 (-1351~-1172)	TTTGGGGTTCTGGCGTCTGG	TGGACAGAGGTGGAGGTGGT	CHIP
STAT3 (-1710~-1530)	TACCTGTAGA GTGTGTGTGT	GGCAGGGAGTGTATAAGGTGTGTA	CHIP
